# Religion and the internet: digital religion, (hyper)mediated spaces, and materiality

**DOI:** 10.1007/s41682-021-00087-9

**Published:** 2021-10-19

**Authors:** Giulia Evolvi

**Affiliations:** Erasmus University Rotterdam, Rotterdam, The Netherlands

**Keywords:** Digital Religion, Space, Materiality, Hypermediated Spaces, Internet

## Abstract

This article offers theoretical reflections on the study of religion and the Internet by critically discussing the notion of “digital religion” (Campbell 2012). In particular, it stresses the importance of integrating material and spatial approaches to the study of digital religion. In doing so, it proposes the theory of “hypermediated religious spaces” to describe processes of religious mediation between online and offline environments by taking into account materiality and space. The article discusses theoretical perspectives by means of case studies: first, the importance of materiality within Internet practices is illustrated through the example of Neo-Pagan online rituals; second, the notion of space, and “third space” in particular, in relation to Internet practices is analyzed through the case of the hashtag #Nous-Sommes-Unis, circulated by French Muslims; third, the theory of hypermediated spaces is exemplified by the analysis of a live-streamed mass in the Italian city of Manerbio during the Covid-19 lockdown. The article aims at kindling scholarly reflections on terminologies and theories for the global and interdisciplinary study of digital religion.

## Introduction

The image of Pope Francis standing alone in a dark and empty St Peter’s square on the 27th of March 2020, giving a special *Urbi et Orbi* blessing to pray for the end of the Covid-19 pandemic (Watkins 2020), exemplifies some facets of religion during the Covid-19 lockdown. The blessing is symbolically important as it occurred when the world was struggling with new lockdown restrictions, particularly severe in Italy. Arguably, the image is remarkable because of the emptiness of St. Peter’s square, a space usually full of believers. As people could no longer physically participate in religious functions, the special *Urbi et Orbi* was consumed exclusively through media, either television or Internet streaming.

This image may suggest that the Covid lockdown brought extraordinary changes to religion, with mediation substituting the physicality of believers gathered around the pontiff. Indeed, during the Covid-19 lockdown several religious communities around the world were forced to find online strategies to substitute physical interactions (Campbell 2020a). However, two considerations need to be done. First, as media scholar Stewart Hoover (2006) asserts, religion and media have always been entangled throughout history. Religion is inevitably based on messages diffused through media, from sacred texts to new media technologies (Horsfield 2015). The second consideration is that the reproduction of religious rituals online is not something that is only caused by physical distancing restrictions, and it is not limited to social network sites (Hadden and Cowan 2000). For instance, Pope John Paul II already spoke favorably about the possibility of diffusing Catholic messages online at the World Communication Day in 1990 (Jonh Paul II 1990). Since then, some religious communities from all traditions chose to partially or completely move their activities to the Internet, creating the phenomenon of “online churches” (Hutchings 2017).

Hence, the possibility of participating in the Pope’s mass through media technology is not something new or extraordinary. However, an image such as that of Pope Francis preaching alone in St Peter’s square is arguably impactful because it makes people aware of the absence of physicality during the function and the empty space around the pontiff. The intensification of online mediation during the Covid-19 pandemic urges scholars to further reflect upon questions they have already asked for decades: how does mediation affect religious communities, identities, and authorities? How do people recreate a sense of materiality through digital technologies? How are spaces of religious practice negotiated online? With these questions as starting point, the aim of this article is to present theoretical reflections on the notion of “virtual environment” in relation to religion. Concerning terminology, in the article I will predominantly use the term “space” instead of “environment.” This is because I draw from theories that define space as the result of material and mental practices (Knott 2014), and help me contextualize space as connected to the Internet. In the first part of the article, I will explain that online and offline spaces are entangled in contemporary religious practices by presenting the theoretical framework of “digital religion” (Campbell 2012). Then, I will discuss the notion of materiality in relation to the theory of mediation (Meyer 2010). I will also offer reflections on the notion of space and the Internet, discussing the spatial turn in religious studies and the notion of third spaces of digital religion (Hoover and Echchaibi 2014). Lastly, I will discuss the theory of hypermediation (Scolari 2015) to describe the fluid interactions between different media platforms and physical spaces. It is important to notice that materiality and space are increasingly incorporated in studies of technologies, media, and related fields (see, for instance, Herzogenrath 2015), so this article is connected to larger scholarly debates on these topics.

While the scope of the article is mainly theoretical, I will illustrate my claims employing some case studies about different religious traditions. In specific, I will describe Neo-Pagan online rituals to explore how digital religion employs materiality to enhance a sense of communal participation. I will also analyze the diffusion of the hashtag #NousSommesUnis (We Are United), spread by French Muslims in the aftermath of the 2015 terrorist attacks in Paris, as an example of how digital religion exists in spaces across different platforms. Then, I will analyze the case of a Catholic church in Italy employing live streaming as a substitute for physical engagement during the Covid-19 lockdown to show how materiality and space are hypermediated during moments of limited physical interactions. This last case is the only one addressing religion during the Covid-19 pandemic, and aims both at highlighting how this historical moment intensified online practices, and at stressing continuities with previous examples of digital religion. These case studies are based on my own research and present data acquired through qualitative interviews and qualitative content analysis of media texts. Establishing whether online religious functions and interactions are authentic is beyond the scope of this article, but it aims at offering an overview of theories on materiality, space, and religion that will hopefully help understand online and offline performances as entangled rather than separated.

## Digital religion: online and offline practices

Because religious groups have employed the Internet since the 1990s, this is also the period when scholars started analyzing empirical cases of religion in Internet venues. While it is a relatively recent field of study, the exploration of religion and the Internet already underwent four waves of research (Hojsgaard and Warburg 2005; Lövheim and Campbell 2017). The first wave, which took place in the 1990s, tends to describe the Internet as a space that is separate from physical reality, and that invites practices unrelated to the offline dimension. This is why scholars employed terms such as “cyberspace” and “cyber-religion” (Brasher 2004), which indicated the Interned as a space “other” than everyday activities. During the second wave of studies on religion and the Internet, scholars started to approach digital spaces in a more nuanced way. In the early 2000s, Christopher Helland (2000) introduced the distinction between “religion online,” indicating established religious groups that use the Internet to enhance their activities, and “online religion,” describing interactive online practices existing mostly, or exclusively, online. This distinction is important because, first, it stresses the existence of religious groups that simultaneously occupy online spaces and offline—material—spaces. Second, it highlights how the Internet can create new types of practices that are intrinsically different from those occurring without the aid of digital technologies.

The third and fourth waves are characterized by the theoretical approach of “digital religion.” Differently from cyber-religion or online religion, digital religion entails that online and offline spaces are entangled. Heidi Campbell (2012) describes digital religion as:

[A]rticulating the evolution of religious practice online, as seen in the most recent manifestations of cyberchurches, which are linked to online and offline contexts simultaneously. “Digital religion” does not simply refer to religion as it is performed and articulated online, but points to how digital media and spaces are shaping and being shaped by religious practice.

Hence, a digital religion approach, which I also assume in this article, considers that there is no clear distinction between religious actions that exist online and those that occur offline. This does not mean that the online and offline spheres are identical, and that people do not distinguish between them, but that the Internet is increasingly entangled with everyday activities. The aforementioned example of Pope Francis preaching in St Peter’s square during the Covid-19 lockdown can be considered an instance of digital religion as it shows how an offline space can be mediated to enhance offline practices. Believers would have been unable to listen and watch Pope Francis doing the *Urbi et Orbi *blessing without media technologies, but at the same time the ritual is based on a material space. Therefore, this shows how media technology creates new conditions for the practice of religion where materiality and space do not disappear, but are present in new forms.

Because of the entanglement of physical and virtual actions that characterize digital religion, the terms “online” and “offline” may not always offer a helpful theoretical distinction. Regarding digital practices, philosopher Luciano Floridi (2015) proposes the term “onlife” as a substitute for the online-offline dichotomy. The notion of onlife indicates the blurring of boundaries between real and virtual, as well as of the distinction between human, machine, and nature. While in this article I still employ the terms “online” and “offline” to describe practices that are connected to the Internet or to physical spaces, I embrace the idea of thinking of religious practices as increasingly “onlife.” Rethinking the terminologies that describe digital religion also helps to account for the contemporary high-tech society where many interpersonal communications occur through digital media, including religious experiences.

However, while contemporary religion largely occurs in contexts of constant mediation, there are two important considerations that need to be done. First, most works analyzing examples of religion and media, including the case studies I present in this article, focus on contexts, such as the North American or European ones, with a high presence of digital media (Hjarvard and Lovheim 2012). Scholars also need to consider that there are locations where Internet connection is scarcer, and that the condition of being onlife depends on economic possibility, access to technology, media education, and skills. Second, while media proliferate in several contexts, there are religious communities that make the conscious choice of not engaging with technology. The work of Campbell (2007) on the so-called “kosher phone” describes how certain ultra-orthodox Jewish communities in Israel work together with developers to create smartphones that only connect to sites deemed appropriate by religious authorities. This is an example of how religious groups can consciously negotiate the boundaries between online and offline spaces even in highly mediated contexts. Religious communities’ agency in employing new technologies has also been explored by Campbell with the notion of “spiritualizing the Internet,” an approach that frames the Internet as suitable for religious engagements (Campbell 2005, p. 2). This theoretical standpoint is further elaborated in analyzing how religious people create meaning through media narratives, in what Campbell defines “technological apologetic” (Campbell 2020b).

Therefore, based on Campbell’s approaches, I propose to think about digital religion not as a given, but as a process where religious groups and individuals adopt—and adapt to—digital technologies depending on their values and possibilities. The case of Pope Francis preaching during the Covid-19 lockdown is an example of how religion can—and must—employ digital technologies in situations that offer few alternatives. To explore the entanglements between online and offline venues, it is relevant to analyze two interconnected elements of religious practices: materiality and space.

## Digital religion and materiality

Conceptualizing digital religion as connected to physical experiences helps to think about Internet practices as material. Hence, the Internet is not an immaterial environment “other” than the material reality, but it is linked to practices that exist in material spaces. It is for this reason that, in this section, I will explain the importance of materiality for the theory of religious mediation, and I will apply it to the case study of Neo-Pagans performing online rituals.

Concerning materiality, Keenan and Arweck (2006) write that “The idea of religion itself is largely intelligible outside its incarnation in material expressions” (pp. 2–3). This approach marks a material turn that rejects the dualism between mind and body, and considers art, music, spatial practices, clothing, and literature as integral parts of the religious experience. According to David Morgan (2016), material practices are not secondary aspects, but they are the very core of religion. Examples of religious materiality include the Muslim female veil, which does not only indicate religious belonging, but symbolizes complex negotiations in terms of ethnic identity and status (Ünal and Moors 2012). Besides, Valentina Napolitano (2019) analyzes the act of Pope Francis to show a wooden cross on the Italian island of Lampedusa, where several migrants arrived in dinghy boats from North Africa. The cross, made of wood from shipwrecked boats, became a powerful material symbol employed to unite people and emotionally move them.

The role of objects in everyday religious experiences suggests that materiality is involved in processes of religious mediation that help frame relations among people and diffuse religious messages. In other words, objects constitute what anthropologist Birgit Meyer (2010) describes as “sensational forms,” which are practices and attitudes that help structure the transcendental. Hence, for Meyer (2006) objects allow people to experience religion by materializing it:

Posing a distance between human beings and the transcendental, religion offers practices of mediation that bridge that distance and makes it possible to experience—and from a more distanced perspective of could say: produce—the transcendental. (p. 13)

Meyer explains mediation with the example of the Catholic icon: even if it is human-made, it is believed to embody the sacred presence, and it can be experienced with the senses, through a gaze, a prayer, or a kiss (*ibidem*). Mediation, a theory elaborated also in relation to media consumption (Martin-Barbero 1993), is here conceptualized as a process that brings believers from immanence to transcendence through religious media. The term “media,” in this context, designates all the objects, performances, and pieces of art that participate in the religious experience, and includes audiovisual media, studied by Meyer in Pentecostal-charismatic churches in Ghana. Drawing, among others, from the work of Meyer, Arjun Appadurai (2015) situates the relationship between mediation and materiality beyond the study of religion, describing as “mediants” all the human and non-human actors that participate in material practices, such as those connected to housing and shelter.

Drawing from these theories of mediation, I propose there are three main reasons to approach digital religion considering materiality. First, digital artifacts and technological devices—smartphones, tablets, computers—are themselves material objects that can embody the religious experience (Campbell and Connelly 2020). Second, digital media may not allow tactile practices, but they facilitate visual culture which, Morgan (2005) writes, also helps to materialize religion by providing a “sacred gaze” connected to the sensors consumption of images. Third, there are practices that help “rematerializing” the digital. As Tim Hutchings (2016) writes, the materiality of the Internet lies in the different affordances of the platforms—including design, aesthetics, coding, and interfaces—as well as in the actions and relations that digital practices make possible. Hutchings employs various examples to illustrate this approach: applications that create the possibility of reading the Bible on a technological device and connect with other believers, printed Bibles linked to online videos to enhance the reading experience, and graves with QR codes that permit people to grieve their loved ones with the help of dedicated websites. These case studies show how materiality can be embedded in Internet practices that activate relationships and identities.

Therefore, it is helpful to think of digital media in material terms because they may help people relate with the transcendental in practices of mediation, they include material technologies and visual aesthetics, and they are embedded in everyday material practices. The case of online rituals can illustrate how materiality and mediation are connected: in exploring Buddhist meditation in virtual environments, Heidi Campbell and Louise Connelly (2020) highlight how people meditate through avatars and digital artifacts that reproduce material objects, while at the same time reproducing practices in the offline space. This is also true for Neo-Paganism, an umbrella term that includes several religious communities that draw from pre-Christian beliefs. These groups share various characteristics and usually accord great importance to rituals and community practices, which they adapted to online environments already in the 1990s (Grieve 1995). While not all Neo-Pagans employ digital technologies, many rely on the Internet to get in contact with like-minded people and create online communities (Renser and Tiidenberg 2020). Douglas Cowan (2005) discusses Neo-Pagan online rituals as creating a placeness experience, and emphasizes that an Internet-mediated experience cannot replicate the sensorial practices that people are supposed to activate in a physical location. However, in my previous work (Evolvi 2020) I argued that Neo-Pagan online engagement, while indubitably different than an offline ritual, is also characterized by material expressions.

An example of how Neo-Pagans embed materiality in online rituals can be found on the website JaguarMoon (www.jaguarmoon.org). Based in the U.S., JaguarMoon is a cyber coven that offers online classes and creates environments for online rituals. I interviewed the founder of the website, and we discussed the peculiarities of online rituals, which are mainly held because Neo-Pagans often do not find many other practitioners in their homeplaces, or they fear the social stigma of disclosing their religion. She explained that online rituals were initially held through chatrooms, and then they started to employ software that allowed people to speak and see each other. According to my interviewee, the principle of online rituals is to focus on visualization and connect with other practitioners through the computer to rise energy. She describes online rituals as follows:

In a physical group when everybody is right in a room, you’re all connected, energy raises the air of your arms, back on your neck, you’re based in it. Online rituals, since you’re alone in a room, really depend on your ability of visualization and to literally imagine yourself connected with other people in the astral. But as far as I’m concerned cyberspace is astral, is a different portion of that plane so you are making an energetic connection with the priestess and the leader, what I do when I’m doing ritual is that I visualize a connection being made with all different people around physically (personal communication, 17 September 2016)

In this quote, the interviewee described the importance of visualization to give a sense of physicality and materiality to an online ritual. In the interview, she also explained some strategies to embed material practices in online rituals, such as creating altars and placing candles near the computer. For example, she described a ritual of abundance where people are encouraged to take an egg, paint some symbols on it with cinnamon and vegetable oil while in front of the computer, and eat it the day after. This practice helps people to feel the material part of the ritual while at the same time being connected with others through the Internet. Materiality is embedded in various ways: the computer is a material object that makes people feel connected with each other, tangible objects such as eggs and candles help believers recreate a sense of materiality around the computer, and imagination allows the participants to visualize the ritual and raise the energy as a community. The interviewee, as per the aforementioned quote, considered cyberspace as an astral place, where it is possible to conduct a ritual. According to this approach, the online rituals enabled by the JaguarMoon community are an example of online mediation as they consider the online connections and the objects around the computer as concurring to help believers attain the transcendental.

Therefore, the embeddedness of online and offline spaces and practices that characterize digital religion also includes processes of mediation based on material objects. In the case of the Neo-Pagan rituals I have described, practitioners shift between online communication and offline materiality and create a new space for the practice of religion. Similarly, when Pope Francis did his *Urbi et Orbi *blessing, the physical and material space of St. Peter’s square was mediated through technology with the aim of bringing a religious ritual in the homes of the believers. While these are two very different examples, they have in common the visual and material mediation of religious spaces through the Internet. In the next section, I will explain how the study of materiality and digital religion also needs to take into account space.

## Digital religion and space

Digital religion is characterized by material practices that happen between online and offline experiences and help to create spaces of community formation and discussion. Therefore, in this section I will contextualize the importance of space and the spatial turn in the study of religion, and I will explain how the notion of space can be applied to Internet practices. I will then analyze how spaces are mediated through different platforms, drawing from the example of #NousSommesUnis, which young French Muslims circulated to challenge stereotypes and condemn religious violence.

The field of religious studies has been increasingly interested in the concept of space to understand certain religious representations and explore the interplay between secular and religious practices. Kim Knott (2014) invites us “to reflect about space as a medium in which religion is situated” (p. 3), not only considering spaces that are “sacred,” but mapping the presence of religion in everyday spaces. From this perspective, space is created through interpersonal relations and embodied experiences, and includes both religious and non-religious manifestations. Knott draws from Lefebvre (1992) to conceptualize space in both material and metaphorical terms. In particular, Lefebvre describes a spatial triad constituted by three dialectically interconnected aspects. First, space can be conceived, meaning that it holds certain pre-planned characteristics that are usually connected to power relations. Second, space can be lived, which means that it may be modified by people’s imagination and their disruptive practices, but it is also connected to traditions and symbols. Lastly, space can be perceived, connected to spatial practices that condition how people use a certain environment through their performances. This triad shows how space has certain aims, but these aims can be subverted by both imaginary and material efforts. Applied to religion, this distinction suggests that religious groups and individuals can negotiate spatial practices and imaginaries to either maintain certain hegemonic and traditional spaces or create new uses for existing spaces.

The work of Kim Knott et al. (2016) on religion in urban environments shows the importance of assuming a spatial approach when studying religion. In urban environments, cultural and religious diversity are both traces of a city’s history and signs of a changing society, and spatial strategies from different religious communities can contribute creating what scholars call post-secular—or “super-diverse”—societies (Becci et al. 2017). For example, Christian churches in Europe are sometimes converted into non-religious places, or they might be used by other religions, such as Islam. The importance of religion in cities shows how the entanglement of religion and space also holds strong material aspects, as these buildings are characterized by material practices of reconversions and the presence of iconic objects that embody religion.

Space and religion are also connected to the diffusion of technology. As Kong (2003) writes, technological developments open up new spaces for the practice of religion, as well as the formation of identities and communities. As technology evolve, space and the Internet can correlate in different ways: on the one hand, as it is the case of Pope Francis’ *Urbi et Orbi*, a physical space can be mediated through technology, and experienced in the private space of believers’ homes; on the other hand, there are spaces of discussion and interactions that can be created online. For instance, Mia Lövheim (2011) analyzes young women’s blogs as “ethical spaces” that exist at the interstice of producers, texts, and users. This ties to larger debates in media studies, which describe the Internet through spatial metaphors (Rogers 2015) or approach spatiality as potentially enabling discursive performances online (boyd and danah 2006). In this context, conceptualizing Internet practices as creating space is helpful to consider how platforms may allow the discussion of social and cultural norms.

Applying the concept of space to media theory, Stewart Hoover and Nabil Echchaibi (2014) conceptualize Internet venues that allow religious expressions as “third spaces of digital religion.” This approach considers religion in broad and fluid terms, incorporating also religious-like practices that develop thanks to the communication potential of digital technologies. Drawing from Homi Bhabha’s (2004) conceptualization of hybrid spaces, Hoover and Echchaibi consider third spaces as generative of hybrid identities and aesthetic imaginaries that derive from the encounter of different online and offline narratives. Hence, even if the Internet might not seem an authentic space for all religious believers, people engaging with third spaces usually consider them as authentic venues for religious practices. Not all online environments hold such characteristics, but third spaces are venues that people approach as-if they are legitimate spaces of religious practice and discussion. For instance, Susanne Stadlbauer (2021) applies the theoretical framework of third spaces to the study of Salafist YouTube videos, conceptualizing them as spaces where they try to recreate a sense of “purity” of the first Islam. Rosemary Pennington (2018) also employs this framework to analyze blogging practices of Muslims living in non-Muslim countries, describing third spaces are described as follows:

If third spaces are sites of practice and negotiation, where connections and identities emerge through interaction, then it would certainly seem that social media hold the potential to facilitate the existence, however ephemeral, of such spaces. (…) It is not as though these negotiations did not exist prior to the advent of social media; social media simply allow us to see these negotiations as they happen. (p. 633)

As the quote highlights, third spaces do not exist entirely online and are not a completely new phenomenon, but they would not be possible without the Internet. Pennington conceptualizes third spaces as environments that allow people to negotiate their identities in ways that are conditioned by the advent of digital technologies. Inspired by these works on digital religion and spatial practices, I employ the term “space” to indicate online venues where religion is discussed. I use space as a concept that includes both mental and material characteristics, as delineated by Lefebvre (1992), and that can be created through performances that blur the boundaries between online and offline spaces.

**Fig. 1:**
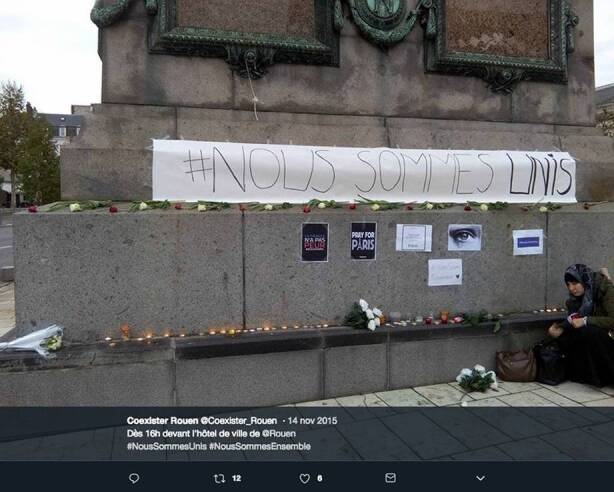
A tweet sent by the association Coexister in Rouen on 14th November 2015

An example of how space is created on the Internet can be found in the case of #NousSommesUnis (We Are United). The hashtag was created in 2015 by the French interfaith association Coexister and used in a video by the student association Étudiants Musulmans de France (EMF, French Muslim Students). I have analyzed the diffusion of the hashtag as an example of how young people engage with digital media (Evolvi 2019). To understand how different spaces concur to create certain discourses, it is useful to trace a brief history of #NousSommesUnis. First, the hashtag was created shortly after the terrorist attacks of the 13th November 2015 in Paris to condemn Islamic terrorism and show that young people from different faiths were united against violence. The hashtag became popular thanks to a video produced and circulated by Muslim members of EMF (EMF Asso 2015) where several people show a sign with #NousSommesUnis, while a voiceover reads a poem expressing grief for the attacks. National and international media outlets discussed the hashtag (see, for example, Buratti 2015). The increasing visibility of the hashtag created multiple venues for people to use it, both online and offline. For example, other people who wanted to show solidarity created videos inspired by the original EMF video, and several individuals and associations used it on social networks also in relation to other attacks and forms of violence. Besides, #NousSommesUnis became an interfaith group with a website (www.noussommesunis.com) used to promote cohesion, collect petitions, and publish people’s testimonies. As Fig. 1 shows, the hashtag has also been displayed during demonstrations against violence. The tweet that embeds the picture reads “At 16 in front of the City Hall of Rouen”, and has the hashtags #NousSommesUnis and #NousSommesEnsamble (We Are Together).

The example of #NousSommesUnis shows how the Internet can offer people possibilities of changing and negotiating spaces. The hashtag enabled young French Muslims, who usually do not have many opportunities to express themselves in the public sphere, a safe space to have their voices heard. This metaphorical space helped change the terms of mainstream conversations, as media reported also this story instead of only focusing on Islamic violence. Moreover, similarly to the blogs explored by Pennington (2018), the website #NousSommesUnis collects people’s voices and becomes a third space where religious identities are discussed. If the practice of circulating online narratives and images can create a space of dialogue, it also has an impact on material spaces. For instance, in the video, #Nous-Sommes-Unis is written on billboards and paper signs and physically displayed by people. Something similar happens in the picture shown by Fig. 1, where the hashtag is used to bring people together in a manifestation of solidarity. These practices, which are based on physical spaces, are then remediated on platforms such as YouTube and Twitter. This example shows how the Internet can embed materiality and space in both metaphorical and material ways.

Therefore, it is helpful to think of case studies such as #NousSommesUnis in terms of materiality and space, and the spatial turn in the study of religion also needs to be applied to digital religion. Digital practices often do not create a single space, but a multitude of online and offline spaces that intersect across platforms, as happened with #NousSommesUnis. It is for this reason that I call these spaces “hypermediated spaces,” to highlight the connections between different platforms, material practices, and digital experiences. In the next section, I will delineate the characteristics of the theory of hypermediation to discuss materiality and space in Internet practices.

## Digital religion and hypermediated spaces

Space and materiality are both relevant aspects of digital religion and contemporary religious practices that exist between online and offline environments. The examples I illustrated in this article show how Internet mediation often occurs on multiple levels and through various interconnected actions. Hence, it is helpful to think of mediation as a complex process that happens across spaces and does not occur through a single medium. In this section, I will present the theory of hypermediation and apply it to a case study on online streaming in an Italian church during Covid-19 lockdown.

Mediation is intensified in online spaces, because the Internet offers venues for rapid connections across the globe. It is for this reason that media scholar Carlos Scolari (2015) describes online communications as examples of “hypermediation.” Scolari draws from the work of Jesus Martin-Barbero (1993), who theorized mediation in communication theory as the practice of creating meaning through communication processes. However, Martin-Barbero’s mediation mainly focused on mass media, and on a type of communication where people receive media messages. Differently, digital communication creates the possibilities for people to participate by creating content and circulate their discourses across platforms. In the aforementioned example of #NousSommesUnis, the hashtag was circulated by various actors who did not only make it reach popularity on the Internet, but also amplified it through so-called mainstream media and employed it in physical spaces.

The notion of hypermediation has been applied to digital religion by the scholars of the Center for Media, Religion and Culture at the University of Colorado Boulder (CMRC 2020). According to this approach (Evolvi 2018), hypermediation is an intensification in emotional responses that elicit social change. In particular, people create religious narratives to rapidly reach like-minded users or to spread fear and anger against those who are perceived as different. In doing so, they create spaces that are between alternative and mainstream narrations, public and private feelings, and real and imaginary religious experiences. It is for this reason that I define “hypermediated religious spaces” those spaces that exist between various platforms, are connected with offline and material experiences, and would not hold the same qualities without the Internet.

**Fig. 2:**
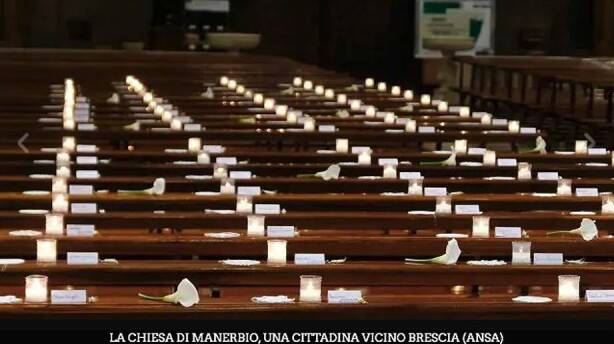
A picture of the live-streamed mass in Manerbio, Brescia. Retrieved from RAI News, https://www.rainews.it/dl/rainews/media/ContentItem-41e0fe7b-5d65-41c6-8d02-385fad4075e5.html#foto-1

During the Covid-19 lockdown, as exemplified by Pope Francis’ *Urbi et *Orbi, Catholic communities had an unprecedented push to digitalization that forced them to find new communicative paradigms (Sabaté Gauxachs et al. 2021). The work of Eline Huygens (2021) on Roman Catholic practitioners during Covid-19 lockdown suggests that people found different strategies to reproduce, or address the lack of, materiality, embodiment, and performativity within digital religion. An example can be found in a live streaming done on the 3rd of May 2020 by the Catholic priest of Manerbio, a town in Northern Italy. The city of Manerbio is situated in the region of Lombardy, the first hit by the Covid virus in Europe, and was among the areas most affected by the pandemic. In spring 2020, the Italian authorities issued a complete lockdown in the country, and people could not attend church functions and celebrate funerals. To address this issue, several priests and religious leaders decided to live stream rituals online. The priest of Manerbio performed a commemorative mass to honor the 150 people from the town that had lost their lives due to Covid-19 since the beginning of the pandemic, and live-streamed it on Facebook. The priest put candles, flowers, and pieces of paper with the names of the decease on the church’s benches, where the believers used to seat, as can be seen in Fig. 2. The mass rapidly gained national resonance (see, for instance, RAI News 2020), as the video and pictures of the empty church with the metaphorical presence of the town’s deceased emotionally moved people across the country.

I consider this an example of hypermediation because it involved different spaces, both enacted through media technologies and embedded in material practices. The physical space of the church, inaccessible because of the Covid-19 lockdown, was mediated through technologies and consumed in people’s private places. Furthermore, the aesthetic of the mass, completed by flowers and candles, was remediated through the Internet, and from there through national television channels and newspapers. This allowed both for the amplification of the event through the live streaming of the space of the church in Manerbio, and for the creation of a space where people could comment, create narratives, and discuss their feelings. Hence, on the Facebook page of the Parish of Manerbio (Parrocchia di Manerbio 2020), where the priest streamed the video and posted pictures of the mass, members of the local community could leave messages offering each other condolences and remembering the deceased. Moreover, several media outlets posted the story on their social networks pages (see, for instance, SkyTG24 2020), attracting comments from people across the country, who often expressed sorrow and solidarity even if they did not have any personal connection with the city of Manerbio. While the case of the Pope’s *Urbi et Orbi *seems similar to the mass in Manerbio—as the space normally filled with believers was empty, and people followed the ceremony through digital technologies—the two created a different model of people’s engagement. The Pope’s mass mirrored a physical mass in St. Peter’s square, with believers being the relatively passive audience of the pontiff. Differently, the priest of Manerbio seemed to invite people’s conversations, as the event was done specifically to involve the members of the local community and give them a space to grief. Therefore, as happened with #NousSommesUnis, the images of the mass in Manerbio rapidly travelled across media platforms also because of people’s engagement in circulating them.

In the case of the mass in Manerbio, materiality also played a role in the creation of hypermediated spaces. As exemplified by the aforementioned case of Neo-Pagan online rituals, objects can enact processes of mediation and help people feel the religious experience. In the live-streamed mass in Manerbio, each candle symbolized a person who passed away because of Covid, metaphorically creating the condition to express grief that did not take place during a funeral. Members of the community did not directly experience the materiality of the candles, but they could experience the symbology of that materiality through the visual mediation of the Internet. This process was characterized by the creation of a space between different venues. First, people experienced the public space of the church, usually associated with town life and community activities, in the private space of their homes. Second, the event was streamed through the Internet to a local community, but it became part of mainstream conversations as it was discussed by various national media. Third, the mass was based on physical space and rooted in materiality, but became effective through the imaginative efforts of people who consumed it through mediation and understood the metaphorical role of candles. Therefore, this can be considered a case of hypermediation not only because it involved space and materiality, but also because it existed at the intersection of various media platforms and spaces.

## Conclusion

This article discussed the notion of digital religion in connection with the material and the spatial turn in the study of religion. In theorizing hypermediated religious spaces, I showed that approaches to religious mediation need to take into account also materiality and space. The examples in this article—Pope Francis’ *Urbi et Orbi *in an empty St. Peter’s square, Neo-Pagan online rituals, the hashtag #NousSommesUnis circulated by French Muslims, and the live-streamed mass in Manerbio—show that contemporary religion is experienced between online and offline spaces, and it is hypermediated in different environments. I would like to offer three concluding remarks on digital religion, materiality, and space.

First, I have replaced the notion of “virtual environments” with “hypermediated spaces” to stress the interconnections between various venues and the meaning-making processes that involve the creation of space. However, I believe that there is a need for further developing the terminology around the notions of materiality and space when it comes to digital religion. If the notion of “onlife” (Floridi 2015) can partially help to highlight the interconnections between online and offline venues, it still needs to be adapted to the negotiation of online religious identities, communities, and authorities. In thinking about terminology, it is important to consider the differences and similarities between types of space: as exemplified by the cases analyzed in this article, some online venues are reproductions of physical ones, while others are live streaming of what happens in a material space, and others are spaces of dialogue and connection. Hence, scholars need to consider digital religion as creating various types of space that are not static, but continuing changing and in conversation with each other, and they need to find theoretical approaches and definitions that reflect these characteristics.

Second, spaces are often defined by material practices, but they are also constituted by mental efforts (Knott 2014; Lefebvre 1992). Therefore, hypermediated spaces do not necessarily reproduce hegemonic spaces, but they also offer the possibility of subverting the status quo by creating new religious imaginaries. Neo-Pagans and Muslims in non-Muslim countries can use the Internet to create communities they do not find in their local environments and establish narratives that are not present in so-called mainstream media. By paying attention to issues of social justice and activisms, the study of digital religion can also explore social inequalities and the creation of alternative spaces. This perspective is also an invitation to look at the creation of spaces outside contexts with a high media usage, and going beyond the North American and European focus that characterizes the study of digital religion.

Third, hypermediation occurs when the Internet allows for fast connections between people in different places, and the circulation of emotional images and narratives. This aspect is intensified when people do not have the possibilities of meeting in person, as happened during the Covid-19 lockdown with the mass in Manerbio. However, if the Covid-19 pandemic forced people to employ certain tools, it did not drastically change communication dynamics. Certain minority groups, such as Neo-Pagans or Muslims in Europe, had already employed online communication because of social marginalization or lack of physical communities. What the Covid-19 lockdown did is make mediation almost inevitable for the experience of religion. While some hypermediated practices might disappear when social and physical distancing rules are no longer in place, the intensification of Internet connections during moments of lockdown likely created the conditions to think of space, materiality, and digital religion in new ways.
